# Improving Asthma Action Plan Completion Rates across Five Divisions in an Academic Children’s Hospital

**DOI:** 10.1097/pq9.0000000000000700

**Published:** 2023-12-05

**Authors:** Maria G. Alfieri, Katie Catalano, Tregony Simoneau, Linda Haynes, Patricia Glidden, Sachin N. Baxi, Ramy Yim, Benjamin Ethier, Faye F. Holder-Niles, Kendall McCarty, Frinny Polanco Walters, Eli Sprecher, Amy Starmer, Jonathan M. Gaffin, Jeffrey Durney, Elizabeth Klements, Brittany Esty

**Affiliations:** From the * Department of Pediatrics, Boston Children’s Hospital, Boston, Mass.; † Division of Pulmonary Medicine, Boston Children’s Hospital, Boston, Mass.; ‡ Division of General Pediatrics, Boston Children’s Hospital, Boston, Mass.; § Division of Immunology, Boston Children’s Hospital, Boston, Mass.; ¶ Division of Hospital Medicine, Boston Children’s Hospital, Boston, Mass.; ‖ Division of Adolescent Medicine, Boston Children’s Hospital, Boston, Mass.; ** Patient Care Operations, Boston Children’s Hospital, Boston, Mass.

## Abstract

**Introduction:**

Asthma is the most common chronic disease among children. Asthma Action Plans (AAPs) enable asthma self-management tailored to each patient and should be updated annually. At our institution, providers face challenges in creating reliable processes to consistently complete AAPs for patients with asthma. This project’s aim was to increase the percentage of patients across five hospital divisions who have an up-to-date AAP from 80% in May 2021 to 85% by October 1, 2021.

**Methods:**

We launched a quality improvement (QI) project using the Model for Improvement, focusing on improving AAP completion rates across five hospital divisions providing ambulatory care for asthma patients. The divisions (Adolescent/Young Adult Medicine, Allergy, Pulmonary, and two Primary Care sites) participated in the QI process using tools to understand the problem context. They implemented a cross-divisional AAP completion competition from June to October 2021. Each month during Action Periods, divisions trialed their interventions using Plan-Do-Study-Act cycles. We held monthly Learning Sessions for divisions to collaborate on successful intervention strategies.

**Results:**

Statistical process control chart analysis demonstrated that the overall AAP completion rate increased from a baseline of 80% to 87% with the initiation of the competition. All divisions showed improvement in AAP completion rates during the active intervention period, but sustainment varied.

**Conclusions:**

The cross-divisional competition motivated five divisions to improve processes to increase AAP completion rates. This approach effectively fostered engagement and idea sharing to boost performance, and may be considered for other QI projects.

## INTRODUCTION

One in 12 children under age 18 has an asthma diagnosis in the United States, making it the most common chronic disease among children.^1^ The risk of morbidity with pediatric asthma is substantial, as the rates of emergency department visits and inpatient hospital stays per 10,000 children with asthma were 108.4 and 8.8, respectively, as of 2019.^2^ Moreover, the prevalence of asthma attacks among children with asthma was 42.7% in 2020.^2^ At our institution, asthma is one of the top diagnoses for inpatient hospital admissions. Although the impact of asthma on patients and families is evident, there are effective means to optimize symptom control.

Asthma Action Plans (AAPs) are an effective communication tool to help families manage asthma symptoms, and the Expert Panel Report 3 Guidelines for the Diagnosis and Management of Asthma recommend that every child with asthma receive a written AAP from their clinician.^3^ For AAPs to be effective, they should be updated with every medication change, annually at a minimum, and shared with patients.^4^ AAPs provide patients and families with a framework for the staged intervention of asthma management at home. They consist of three treatment categories based on symptoms: green (every day), yellow (first sign of illness), and red (urgent).^5^ Studies have shown that AAP creation and distribution positively influence patients’ abilities to self-manage and care for their asthma symptoms, as AAPs facilitate active involvement in asthma management.^6^ They help patients “recognize and respond appropriately to worsening asthma.”^7^ Randomized trials have found that asthma self-management education is associated with reduced hospitalizations, emergency room visits, unscheduled healthcare visits and outpatient revisits, and days off from work or school.^8,9^ Everyone diagnosed with asthma should have a written AAP with information about how to manage symptoms.^5,7,10^

Although AAPs have proven beneficial for asthma care, challenges with clinicians updating them regularly is common. According to the Centers for Disease Control and Prevention, only 46.5% of children with asthma nationwide have ever received an AAP as of 2018.^11^ AAP completion challenges in large academic centers include provider time constraints, poor confidence in provider ability to create AAPs, trouble finding resources to print AAPs, opaque documentation about prior AAP counseling, and availability of staff who can develop AAPs.^12,13^ At our institution, additional barriers included a lack of standard processes to know when patients are due for an AAP and suboptimal technology for efficient creation of AAPs. This quality improvement (QI) project sought to improve our AAP completion rate through a cross-collaborative approach involving sharing learnings between divisions.

### Specific Aim

Our SMART aim was to increase the percentage of patients across five hospital divisions with an up-to-date AAP from 80% in May 2021 to 85% by October 1, 2021.

## METHODS

### Context

We completed this initiative in the Department of Pediatrics at Boston Children’s Hospital, a large urban academic children’s hospital. We included five ambulatory care divisions participating in treating asthma patients: Adolescent/Young Adult Medicine (AYAM), Allergy, Pulmonary, and two Primary Care sites (main-campus and community-campus). The team focused on ambulatory AAP completion because inpatient completion consistently exceeded the target, with a 93.4% mean AAP completion rate over six consecutive years. All divisions completed AAPs in the electronic medical record (EMR), with the date visible to all providers. Each division had its own structures and resources for creating AAPs. During our baseline period (January–May 2021), the mean completion rate was 80%, with upper and lower control limits of 91.13% and 69.61%, respectively. We chose a 5% increase for our aim statement because it is traditionally challenging to improve an already high percentage.^14^ Similarly, each division began at varying starting points, so 85% was an achievable goal for all.

### Interventions

We assembled a multidisciplinary working group with key stakeholders for a QI project. The team included representation from all five divisions and consisted of physicians, nurses, administrative staff, data analysts, QI experts, and an information technology representative. We timed the project to start in the summer of 2021, as there is a need to complete AAPs before children return to school in the fall.

**Fig. 1.**
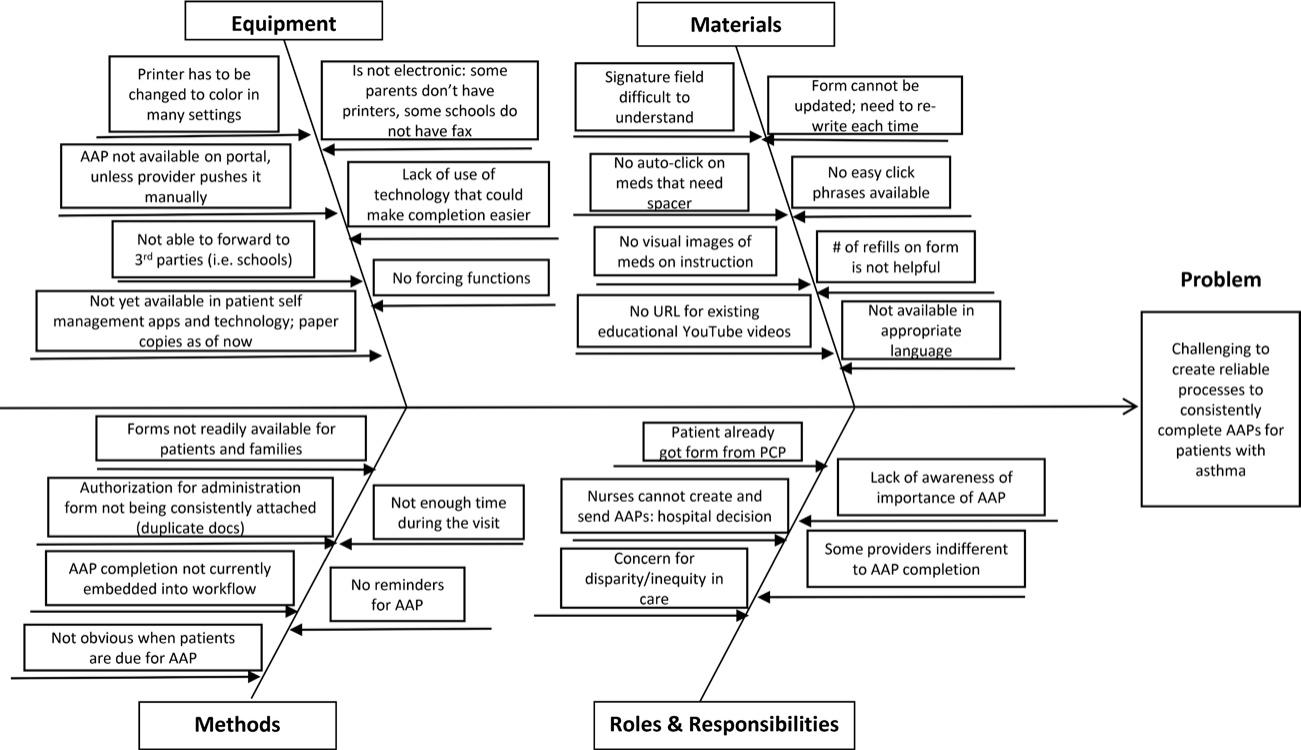
Fishbone diagram outlining barriers to AAP completion; PCP, Primary Care Physician.

**Fig. 2.**
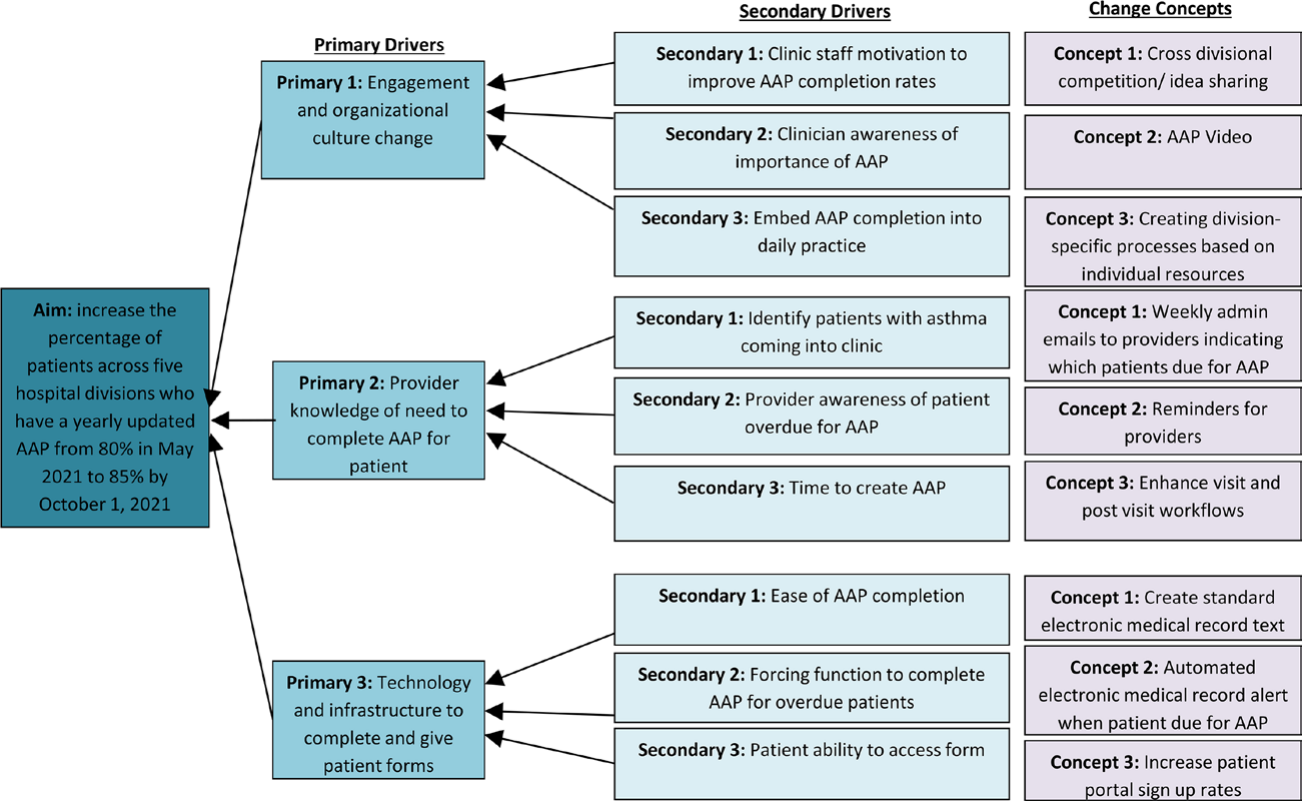
Driver diagram depicting the relationship between the aim, primary drivers, secondary drivers, and associated change strategies.

We framed the project using the Model for Improvement.^15^ Using QI tools, the team completed a current state analysis to understand the causes for why AAPs were not reliably completed. We used process mapping to identify the current state process and to understand the complexities of creating an AAP. Next, the team created a fishbone diagram to conceptualize the main barriers to the problem (Fig. 1). We then developed key drivers and associated change strategies and used a driver diagram to establish the causal pathway between our problem drivers and the intervention selected (Fig. 2). The working group found the three main drivers were: (1) engagement and organizational culture change, (2) provider knowledge of the need to complete an AAP for a patient, and (3) technology and infrastructure in place to create the AAP. Due to the differing resources of each division, we decided to focus this project on the engagement and culture change driver. To enhance engagement, the working group recognized that a clear secondary driver was staff motivation.

**Fig. 3.**
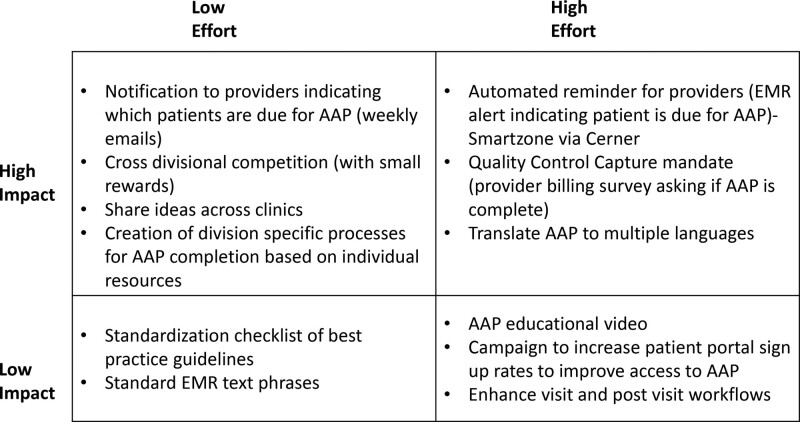
Impact Effort Matrix displaying the team decision-making process for prioritizing intervention ideas based on the expected level of impact and the amount of effort it may take to implement.

We then used an impact effort matrix to decide which change strategies to use (Fig. 3). The primary change strategy selected was a competition between the five divisions. Studies have shown that competition improves performance; so we tested this strategy between hospital divisions.^16,17^ The competition consisted of monthly emails to celebrate the leader(s) and four monthly Learning Sessions where divisions shared successful intra-disciplinary strategies modeled and adapted from the Institute for Healthcare Improvement Collaborative Model for Achieving Breakthrough Improvement.^18^ During Action Periods between Learning Sessions, each division used the Model for Improvement to identify and test their own interventions using Plan-Do-Study-Act cycles.^15,18^ This approach accommodated differences in clinic workflows and supported improved performance in a collaborative learning setting.

Our working group planned the competition logistics and advertised the launch date and details to divisional asthma leaders through email and division meetings. The five divisions trialed their own interventions to improve AAP completion rates to accommodate local differences in capabilities and resources.^19^ The competition reset each month and lasted for 4 months. At the end of each month, we announced the winning division(s) via email and displayed each division’s monthly performance on a bar chart. Divisions were recognized as winners if they were the most improved group from the previous month, the highest performer of the month, or met the 85% target. The Vice Chair of Quality and Outcomes sent congratulatory emails to the winning division’s chief and associate division chief to recognize the progress.

The working group convened for Learning Sessions at the end of each month, and divisional representatives shared successes and challenges regarding interventions trialed during Action Periods in their independent areas. Divisions decided which strategies to adopt based on their individual needs and resources. Pulmonary asthma leaders implemented direct communication with providers with lower AAP completion rates, division-wide education, pre-visit emails specifying patients needing an updated AAP, and division-wide updates at faculty and asthma team meetings. AYAM focused their interventions on frequent communication with providers via email and at division meetings, and mailing letters to patients/families in preparation for the school year. Allergy leaders implemented email reminders, communication with providers with low AAP completion, and divisional meeting updates. Primary Care sites implemented reminder emails sent to providers the evening before their clinic, regular data pulls to create lists of upcoming patients, visual reminders in clinic, and patient letters to encourage AAP use. The multidisciplinary team created standardized EMR text phrases to make AAP completion more efficient. Local quality teams iterated intervention strategies multiple times throughout the project period. Divisions learned from one another and adapted shared strategies in a way that worked for their particular clinic flow. The team planned for sustainment throughout the QI project.

### Measures

The primary measure for assessing the impact of the intervention was the AAP completion rate across the divisions. We chose this process measure because our goal was to improve AAP completion in a cross-collaborative setting. The denominator was the total number of patients in each division with a primary diagnosis of asthma based on billing code (intermittent, mild persistent, moderate persistent, severe persistent, exercise-induced bronchospasm, cough variant, unspecified, and other asthma) in the divisions of Pulmonary and Allergy, or with a top three diagnosis of asthma in the divisions of AYAM and Primary Care (two sites). The denominator only included patients who had a clinic visit via telehealth or in-person. Exclusions included patient care visits for the administration of biologic medications for treatment of asthma. The numerator was the number of patients with a signed AAP in the EMR dated within 13 months before or within one month after the date of service. This measure was well established at our institution and had previously been validated through chart review to ensure accurate capturing of the numerator and denominator. During the project, the data analyst monitored the AAP completion rate weekly and shared findings with the project group monthly. We tracked divisional-level data using the same measurement criteria. As this project occurred during the height of COVID-19, asthma-related emergency department visits, and inpatient admissions were at an all-time low.^20^ Therefore, we did not include these established outcome measures as part of the current project.

### Analysis

We completed the analysis using standard control chart rules. We recognized significant changes as shifts in the mean, using the established eight-point rule for identifying special cause variation.^21^ We defined a 5-month baseline period (January–May 2021). We annotated the Learning Sessions on the chart to identify the impact on our data. The intervention period began at the competition start, June 1, 2021. SQCPack version 7.0 (PQ Systems, Dayton, Ohio) created p-charts for all measures. This project was conducted according to local institutional standards for QI initiatives; therefore, IRB approval was not obtained.

## RESULTS

**Fig. 4.**
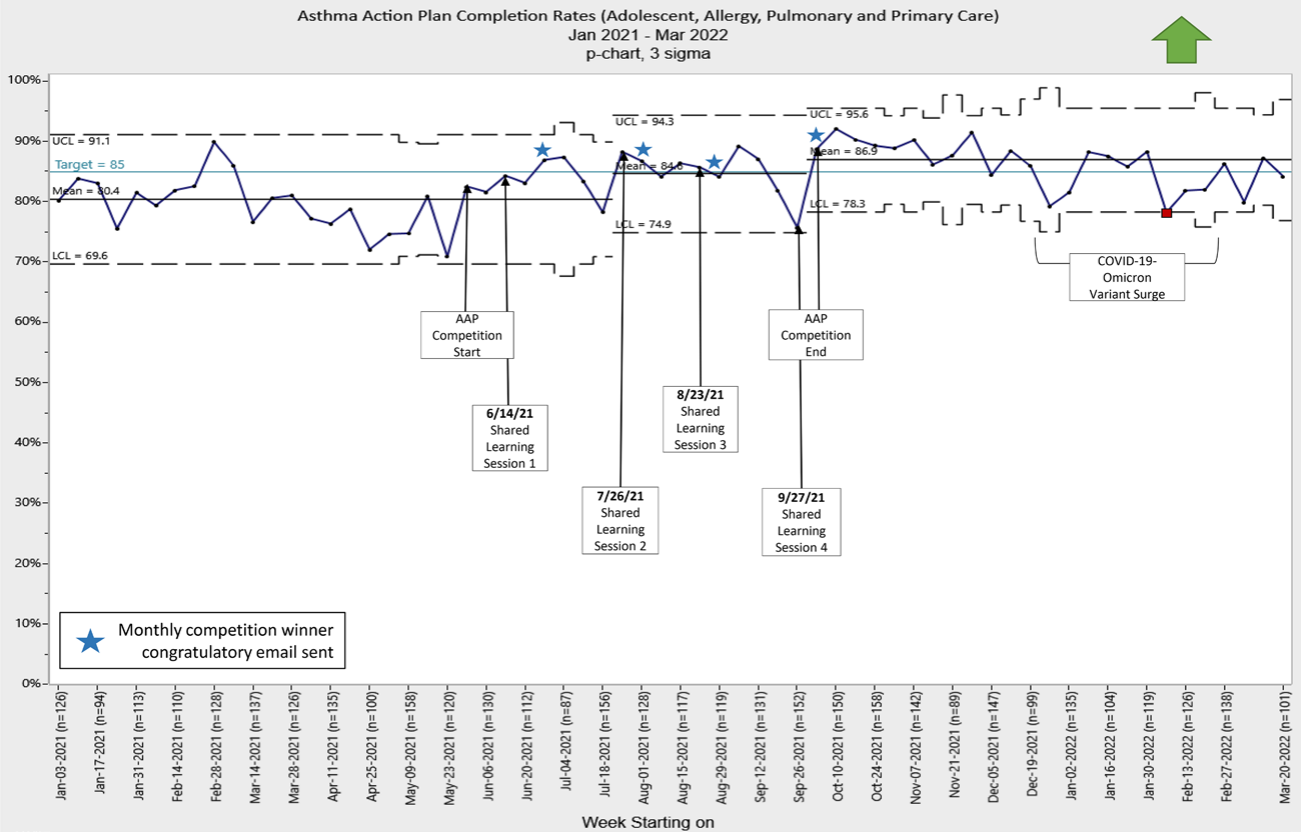
Overall AAP completion rates. P-chart showing the Asthma Action Plan (AAP) completion rate over time. Each point represents the weekly rates of patients from Adolescent (AYAM), Allergy, Pulmonary, and two Primary Care sites which had a signed AAP in the electronic medical record dated within 13 months before or 1 month after the date of service. A solid black line represents the mean rates of AAP completion. The dotted lines denote the statistical upper and lower control limits. There is an upward arrow indicating higher is better. UCL, upper control limit; LCL, lower control limit.

After the competition awareness campaign, there was an upward trend in AAP completion rates. Statistical process control chart analysis demonstrated special cause variation with a shift in the mean AAP completion rate from a baseline of 80%–84% after the first month of sharing learnings and announcing the first winner via email (Fig. 4). There was another shift to 87% upon competition completion. Overall, the AAP completion rate increased from a baseline of 80%–87% with the initiation of the AAP competition, exceeding the 85% target. We had sustainment in the postintervention period with a slight decrease due to resource challenges related to the SARS-CoV-2 omicron variant of COVID-19. Attributing the shifts to a single intervention is difficult, as each division trialed local improvements monthly through internal Plan-Do-Study-Act cycles.

**Fig. 5.**
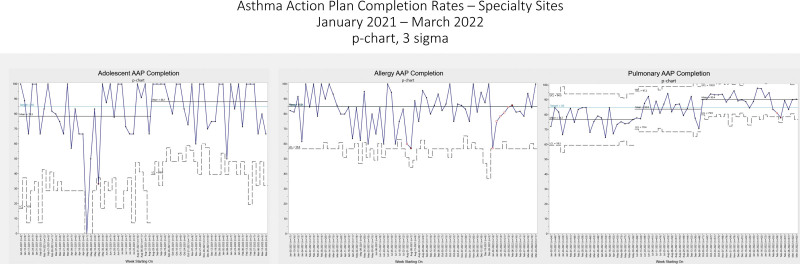
AAP Completion Rates—Specialty Sites. MultiChart consisting of p-charts showing the Asthma Action Plan (AAP) completion rate over time for the divisions of Adolescent (AYAM), Allergy, and Pulmonary. The competition start was June 1, 2021 and the competition end was October 1, 2021 for each division. In each chart, the points represent the weekly rates of patients from the specified division which had a signed AAP in the electronic medical record dated within 13 months before or 1 month after the date of service. A solid black line represents the mean rates of AAP completion. The dotted lines denote the statistical upper and lower control limits. There is an upward arrow indicating higher is better. The open red squares are leading up to special cause variation and the closed red squares mean that special cause variation has occurred. UCL, upper control limit; LCL, lower control limit.

**Fig. 6.**
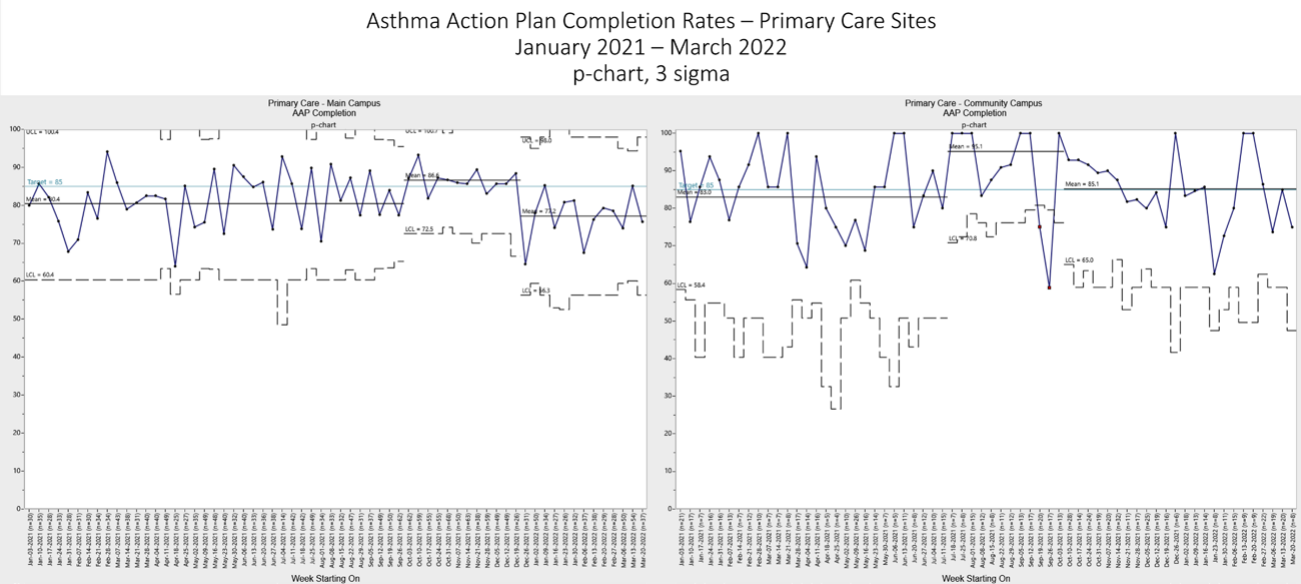
AAP Completion Rates—Primary Care Sites. MultiChart consisting of p-charts showing the Asthma Action Plan (AAP) completion rate over time for the divisions of Primary Care Main Campus and Primary Care Community Campus. The competition start was June 1, 2021 and the competition end was October 1, 2021 for each division. In each chart, the points represent the weekly rates of patients from the specified division which had a signed AAP in the electronic medical record dated within 13 months before or 1 month after the date of service. A solid black line represents the mean rates of AAP completion. The dotted lines denote the statistical upper and lower control limits. There is an upward arrow indicating higher is better. The open red squares are leading up to special cause variation and the closed red squares mean that special cause variation has occurred. UCL, upper control limit; LCL, lower control limit.

On the divisional level, the mean AAP completion rates were as follows in the baseline period (January–May 2021): AYAM, 78.52%; Allergy, 84.95%; and Pulmonary, 76.75% (Fig. 5); Primary Care community-campus, 82.96%; and Primary Care main-campus, 80.38% (Fig. 6). After the intervention, the AYAM AAP completion rate shifted significantly to 88.14%, and was sustained. After further interventions, the Pulmonary AAP completion rate significantly shifted to 83.79% soon after initiation and shifted again to 90.42%. Although Pulmonary experienced a decline coinciding with the SARS-CoV-2 omicron variant circulation, high completion rates were later sustained. Allergy had a decline in August 2021 due to tempoary resource constraints; however, there was an upward trend indicating special cause in the sustainment period. Primary Care community campus initially had a significant mean shift to 95.15% after the start of the intervention. The mean declined to 85.14% after the competition ended, but still met target. Primary Care main campus followed a similar trend, shifting initially to 86.57% with the initiation of the intervention and then decreasing to 77.17% months after the competition ended.

## DISCUSSION

### Summary

Using a competition approach to enable shared learnings, we improved AAP completion rates from 80% to 87% throughout the project period, exceeding our target. All divisions showed improvement in AAP completion rates during the active intervention period, while sustainment varied. There are several strengths of this project. First, the competition enhanced motivation and encouraged rapid-cycle change. Divisions were eager to improve and used the phased structure to adapt, adopt, or abandon local change strategies throughout the 4-month competition. Second, the learning setting fostered creative idea-sharing between divisions. Each month during Learning Sessions, divisions recounted the successes and challenges of the interventions they trialed. Successful strategies were tested and adopted by multiple divisions. Although we cannot identify which interventions led to improvement, divisions reported benefits in strategies focused on raising awareness of AAPs, identifying patients needing updated AAPs before their appointments, and communicating with providers with low AAP completion rates. Third, leadership support incentivized divisions to participate. All asthma staff received recognition when their division was a monthly winner, which created excitement for all asthma staff aside from the working group team only.

### Interpretation

We believe the AAP competition increased awareness, agreement, adoption, and adherence to strategies to improve AAP completion rates. Using QI tools to understand the problem, the working group found that engagement and organizational culture change were the primary drivers for improving the AAP completion rate. We determined clinic staff must be motivated for change, and the competition approach was a successful strategy to drive improvement in this context. The Awareness-to-Adherence Model states that for clinicians to comply with recommended care guidelines, such as AAP completion, they must first be aware of the guidelines.^22^ We believe the competition advertisement highlighted the importance of AAP completion to clinicians, generating awareness. Next, clinicians must agree to complete up-to-date AAPs for all patients with asthma. The interventions that simplified creating AAPs, such as emailing weekly lists of patients overdue and using standard EMR text, eased the burden of knowing when and how to start an AAP, enabling agreement. The next stage of the model is adoption, which we believe played a role toward the end of the competition when clinicians became accustomed to the AAP interventions introduced into practice.

Last, clinicians welcomed interventions to sustain adherence to AAP completion. Post-competition, the QI team continues to share divisional-level AAP completion rates with asthma divisional representatives via email, including the Vice Chair of Quality and Outcomes. Leaders then share data with divisional teams celebrating wins and further encouraging improvement. We believe this has continued fostering awareness of AAP completion within and between divisions, building awareness and motivation to maintain and improve rates. Pulmonary experienced the most robust sustained improvement, while Primary Care main campus did not sustain improvements after the competition concluded. Challenges in sustainment included the need to train new pediatric residents each year and the administrative demands of back-to-school paperwork for providers, both of which the division is aware of and views as an opportunity for improvement.

Our results add to the existing literature around AAP completion. A past study found that healthcare professionals in a primary care setting infrequently review and update AAPs with patients, leading to outdated AAPs and a lack of interest by the patient to use the AAP.^23^ This study concluded that breaking the cycle of ineffective AAP implementation would require a systems approach with multifaceted interventions addressing patient, professional, and organizational barriers.^23^ However, our project shows that fostering an environment welcoming change while leaving the intervention strategies up to the local areas can also be successful. Divisions thrived in a setting where they could use local change strategies. Similarly, another study found that creating an evidence-based guideline to help create yellow zone AAP recommendations increased AAP completion by clinicians.^13^ That study addressed the barrier of provider-level knowledge of the tool, while our project builds upon the findings to address challenges, including clinician resources to create AAPs. To our knowledge, our project is the first time that an organized competition was used as a method to work toward a clinical QI goal. Public reporting has been shown to increase competition and thus motivation to improve between hospitals.^16,17^ Our project mirrors this approach inside an organization through transparency of AAP rates between divisions to increase engagement.

This QI project substantially impacted the care of our patients with asthma, increasing the percentage of patients in our practice who received an up-to-date AAP. AAPs are important for patients and their families to understand how to manage their asthma.

### Limitations

Our initiative has several important limitations. First, results may not be generalizable as the project took place at a single institution with a unique hospital division structure. Second, while we selected a SMART aim at 85% as we felt was realistic and attainable for each division, AAPs are recommended for all patients. Future work will aspire for all patients with asthma to have an updated AAP. Additionally, the denominator only includes patients who had a clinic visit in which asthma was billed as the primary (Pulmonary and Allergy) or a top three (AYAM and Primary Care) diagnosis, which may have excluded some patients with asthma. Furthermore, we did not address whether clinicians taught AAP content to families, which is an area to consider for a future project around asthma education. Last, the extent to which we will sustain performance is unclear, as resources to complete AAPs depend on provider time and availability. We hope to mitigate this by integrating automatic solutions to AAP completion in the future. We are working with our information technology teams on the implementation of technological advances, including automated medical record reminders when AAPs are close to expiration.

### Conclusions

The cross-divisional competition was associated with the improvement of AAP completion rates across the five divisions. Leadership support, celebration of accomplishments, and the collaborative learning setting may have boosted performance. The competition was influential in fostering engagement and idea-sharing. We believe a friendly, supportive competition approach can be considered in other QI projects without a one-size-fits all intervention strategy.

## DISCLOSURE

The authors have no financial interest to declare in relation to the content of this article.
